# Eclampsia after Labor

**Published:** 1895-10

**Authors:** D. A. York

**Affiliations:** Cornelia, Ga.


### ECLAMPSIA AFTER LABOR.

By D. A. YORK, M.D.,

Cornelia, Ga.

I will submit to you for publication a case of eclampsia that occurred eight hours after labor.

I was called to attend Mrs. B. A., primipara, in July, 1895. I found os dilated and head presenting. I saw that her feet and legs were very much swollen, so I gave her thirty grains chloral hydrate in about thirty minutes' time. Labor progressed rapidly and was terminated without any accident. I removed placenta after a rest of ten or fifteen minutes. I gave the nurse directions about using the bichloride douche, and then I departed. I returned at noon and found patient in good condition and very lively. I went away feeling elated over the case and her seemingly fair condition. At 3 P. M. I was summoned with all haste to her bedside, and found her in a convulsion. I have never witnessed such violent spasms in all my professional career. I gave her $\frac{1}{4}$ gr. morphine and $\frac{1}{150}$ gr. atropine hypodermatically and 30 grs. chloral hydrate per rectum, and I gave her another hypodermic of $\frac{1}{4}$ gr. morphine and $\frac{1}{150}$ gr. atropine. In a minute or two she was having another convulsion. It looked as if what I had done was futile. I then changed my treatment. I gave her 3 minims of Norwood's tincture of veratrum viride hypodermatically, and in twenty or thirty seconds I gave her two more minims. When I commenced giving the veratrum she was having a convulsion, and I gave her another injection of veratrum, three minims, hypodermatically. She never had another spasm, only just an effort. Dr. S. came in shortly, and for fear of returning convulsion we gave her per rectum 30 grains pot. bromide and chloral hydrate each.

I had to use the catheter once, then the kidneys performed their functions well. I examined the urine and found a good trace of albumin. The patient made a rapid recovery.

I give this case of eclampsia because I attribute to veratrum the first place in the treatment, and, again, because of the time from the birth of the child to the attack of convulsions.

